# Supplementary material for: The interactome of intact mitochondria by cross-linking mass spectrometry provides evidence for coexisting respiratory supercomplexes
Source: Mol Cell Proteomics. 2017 Dec 8;17(2):216–32. doi: 10.1074/mcp.RA117.000470 (PMC5795388; doi:10.1074/mcp.RA117.000470)
Supplement: Supplemental Data [file supp_RA117.000470_133922_0_supp_23978_fzffwf.zip › spectra_annotation/mito_spectra_annotation/363-1-18-1-16-1.pdf]

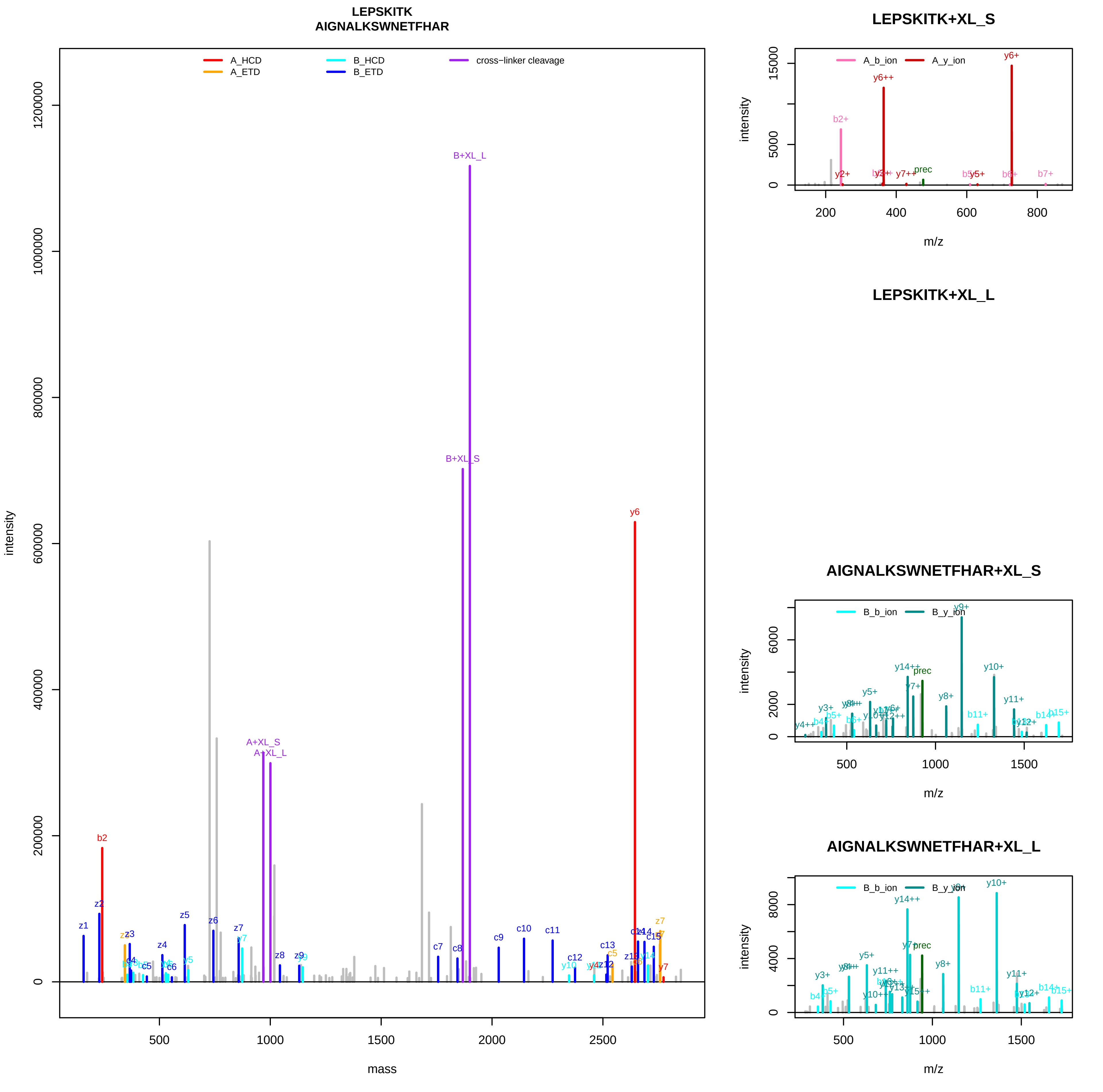
LEPSKITK
AIGNALKSWNETFHAR
A_HCD
A_ETD
B_HCD
B_ETD
cross−linker cleavage
B+XL_L
B+XL_S
A+XL_S
A+XL_L
y6
b2
intensity
mass
0
200000
400000
600000
800000
1000000
1200000
500
1000
1500
2000
2500
LEPSKITK+XL_S
A_b_ion
A_y_ion
y6+
y6++
b2+
prec
y2+
y7++
y5+
b7+
m/z
0
5000
15000
200
400
600
800
LEPSKITK+XL_L
AIGNALKSWNETFHAR+XL_S
B_b_ion
B_y_ion
y9+
y14++
prec
y10+
y5+
y7+
y8+
y11+
y3+
b11+
b15+
0
2000
6000
1000
1500
AIGNALKSWNETFHAR+XL_L
y10+
y14++
y8+
y11+
b11+
b15+
0
4000
8000
